# Between the Baltic and Danubian Worlds: The Genetic Affinities of a Middle Neolithic Population from Central Poland

**DOI:** 10.1371/journal.pone.0118316

**Published:** 2015-02-25

**Authors:** Wiesław Lorkiewicz, Tomasz Płoszaj, Krystyna Jędrychowska-Dańska, Elżbieta Żądzińska, Dominik Strapagiel, Elżbieta Haduch, Anita Szczepanek, Ryszard Grygiel, Henryk W. Witas

**Affiliations:** 1 Department of Anthropology, Faculty of Biology and Environmental Protection, University of Łódź, Łódź, Poland; 2 Department of Molecular Biology, Medical University of Łódź, Łódź, Poland; 3 Biobank Lab, Department of Molecular Biophysics, Faculty of Biology and Environmental Protection, University of Łódź, Łódź, Poland; 4 Department of Anthropology, Faculty of Biology and Earth Sciences, Jagiellonian University in Kraków, Kraków, Poland; 5 Museum of Archaeology and Ethnography in Łódź, Łódź, Poland; Estonian Biocentre, ESTONIA

## Abstract

For a long time, anthropological and genetic research on the Neolithic revolution in Europe was mainly concentrated on the mechanism of agricultural dispersal over different parts of the continent. Recently, attention has shifted towards population processes that occurred after the arrival of the first farmers, transforming the genetically very distinctive early Neolithic Linear Pottery Culture (LBK) and Mesolithic forager populations into present-day Central Europeans. The latest studies indicate that significant changes in this respect took place within the post-Linear Pottery cultures of the Early and Middle Neolithic which were a bridge between the allochthonous LBK and the first indigenous Neolithic culture of north-central Europe—the Funnel Beaker culture (TRB). The paper presents data on mtDNA haplotypes of a Middle Neolithic population dated to 4700/4600–4100/4000 BC belonging to the Brześć Kujawski Group of the Lengyel culture (BKG) from the Kuyavia region in north-central Poland. BKG communities constituted the border of the “Danubian World” in this part of Europe for approx. seven centuries, neighboring foragers of the North European Plain and the southern Baltic basin. MtDNA haplogroups were determined in 11 individuals, and four mtDNA macrohaplogroups were found (H, U5, T, and HV0). The overall haplogroup pattern did not deviate from other post-Linear Pottery populations from central Europe, although a complete lack of N1a and the presence of U5a are noteworthy. Of greatest importance is the observed link between the BKG and the TRB horizon, confirmed by an independent analysis of the craniometric variation of Mesolithic and Neolithic populations inhabiting central Europe. Estimated phylogenetic pattern suggests significant contribution of the post-Linear BKG communities to the origin of the subsequent Middle Neolithic cultures, such as the TRB.

## Introduction

Since the publication of works by Menozzi et al. [1] and Ammerman and Cavalli-Sforza [2] on variation of classical genetic markers in modern-day Europeans, the Neolithic transition has been thought to be one of the most important demographic events in the peopling process of Europe which has followed the arrival of the anatomically modern *H*. *sapiens* in the Upper Paleolithic. The authors estimated that nearly 30% of the variation of the markers reflects a gradient running from the southeast to the northwest, corresponding to the direction of the spread of the Neolithic across Europe from the primary center of Neolithization in the Near East, as confirmed by radiocarbon dating. Although this genetic cline does not have a temporal scale (and may also result from processes other than demic movements, as was suggested by some researchers [3]), its remarkable agreement with the archaeological findings and their radiocarbon dating as well as with other genetic evidence presented in numerous subsequent works [4–10] seemed to support the idea that a new type of economy had been brought to Europe through large-scale migration of the first farmers from the region of Levant/Anatolia, which fundamentally changed the genetic structure of the continent’s population (but see also [11–13]).

Currently, the main source of information on the impact of the Neolithic transition on the genetic structure of Europe is data provided by ancient DNA, and especially mtDNA analysis, which is much more abundant in human remains. Recent studies have shown that the first farmers in central Europe, belonging to the archaeological LBK culture, which emerged in the mid-6th millennium BC in the area of present day Transdanubia, Slovakia, Austria, and the Great Hungarian Plain, and soon spread to many parts of central Europe, initiating there the Neolithic revolution,were allochthonous populations that considerably differed from the indigenous Mesolithic foragers [14], but shared an affinity with the modern-day Near East and Anatolia [15]. While archaeogenetic studies of these two groups of people have clarified one of the central issues concerning the Neolithic revolution, i.e. how agriculture came to central Europe, they have also given rise to other questions due to the fact that the modern inhabitants of this part of the continent cannot be traced back to them. This lack of continuity between either LBK farmers or Mesolithic hunter-gatherers and modern populations in central Europe indicates that the formation of the genetic structure of human populations in this region was greatly affected by demographic processes (migration and admixture, assuming the absence of natural selection acting on particular mtDNA lineages) which followed the arrival of the first farmers [14, 16]. Of special interest is the relationship between the first LBK farmers and indigenous foragers in the subsequent stages of the spread of the Neolithic in central Europe. What was the extent of LBK farmers’ migration over this part of the continent? Was it a one-off event after which groups of farmers were absorbed by autochthonous populations, which quickly adopted the Neolithic economy and technology, or a long-time and recurrent influx of many waves of allochthonous populations that came to dominate the indigenous foragers? How were the relations in the area of LBK colonization affected by the local biogeographic conditions and the degree of sedentism of the Mesolithic foragers?

Recently, Brandt et al. [17] presented a very comprehensive analysis of the formation of mitochondrial genetic variation in skeletal populations from the Mittelelbe-Saale region in central-east Germany, which sheds some light on demographic changes in central Europe since the onset of the Neolithic until the Early Bronze Age. According to the authors, the mtDNA haplogroup composition of the first farmers (LBK) remained stable in central Europe for approximately 2500 years from the moment of their arrival in that area (about 5500 BC). One of the characteristic features of these Early Neolithic cultures was a high frequency of haplogroup N1a with the occasional occurrence of U lineages (typical of hunter-gatherers), which in general persisted through the Middle Neolithic. This genetic continuity was disrupted approximately 3100 BC by an influx of hunter-gatherer haplogroups from the north [17]. Similar results were obtained from a detailed analysis of haplogroup H variation, also based on archaeogenetic data from the Mittelelbe-Saale region [18]. In this case too, the lack of continuity between the LBK and the later, Middle Neolithic cultures indicates a major genetic transition that occurred about 4100–2200 BC, when the mtDNA lineages of the first farmers were largely superseded.

**Fig 1:**
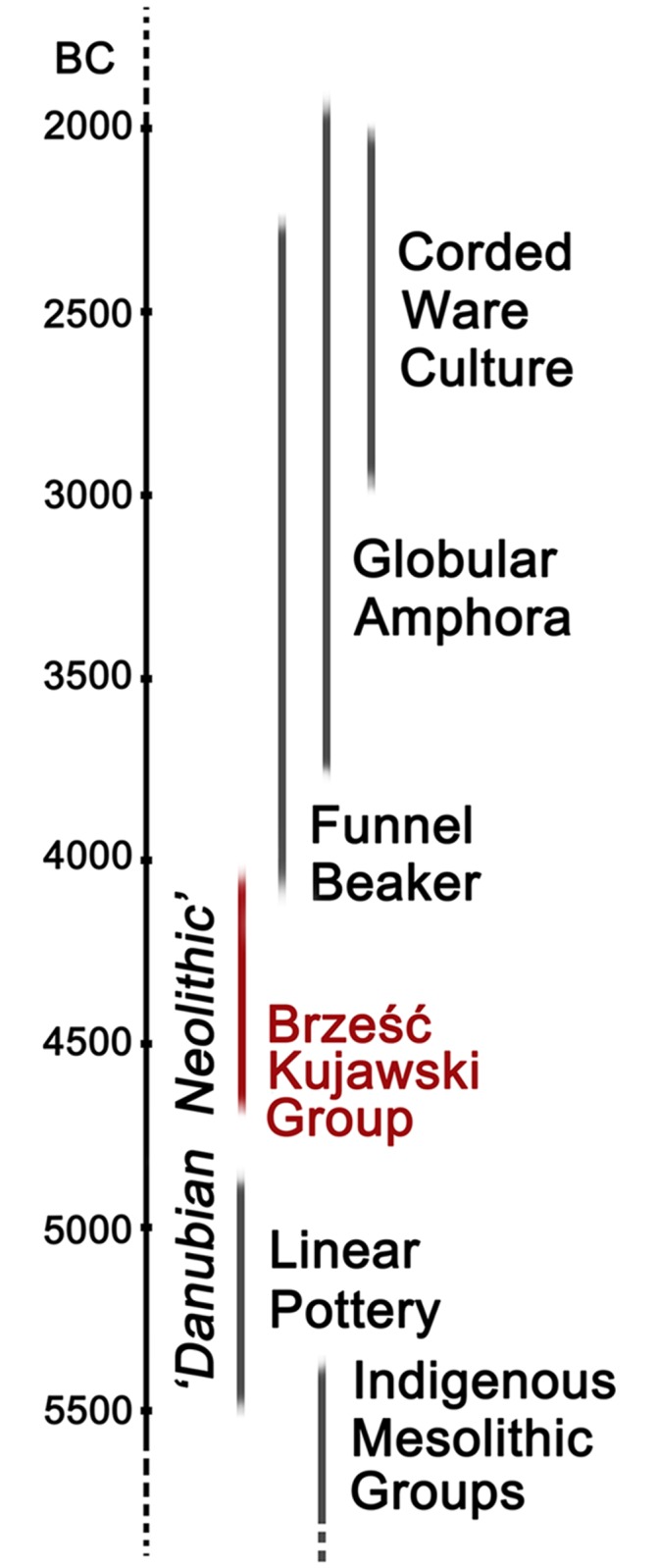
Chronological chart showing major cultural units in Kuyavia region in the period between 5500 and 2000 BC (after: [28, 53]).

The Mittelelbe-Saale region of Saxony-Anhalt in Germany provides exceptional opportunities for the study of ethnogenetic changes due to the access to large number of skeletal series documenting the continuous population of this area throughout the Neolithic and the Bronze Age, which has also been of great interest to anthropologists over the past several decades [19]. However, the question arises as to whether the pattern of changes established for that region is of universal nature, especially in light of many recent works (based on, e.g., craniometric data) suggesting considerable local variation in the Neolithization process of Europe [20–22]. This problem seems essential particularly in respect to the regions which remained borderlands between Early and Middle Neolithic farmer communities and indigenous foragers for a long time [23]. One of such regions is Kuyavia in north-central Poland, where the first LBK farmers arrived as early as about 5500 BC, and then, after a short period of depopulation at the turn of the fifth millennium BC (reflecting the general demographic decline of farmer communities in central Europe at that time [24–26]), their populations stabilized during the fifth millennium BC within post-Linear Pottery cultures, such as the Stroke-Ornamented Pottery culture, and particularly the Brześć Kujawski Group of the Lengyel culture (BKG) [27] (Fig. 1). For over one millennium these post-Linear farmer communities constituted the border of the “Danubian World” in this part of Europe, probably keeping some contacts with also relatively sedentary foragers of the North European Plain and the Baltic coastal zone [23, 28, 29]. The nature of those contacts (trade or perhaps a flow of members of one community to the other, e.g., forager women to the farmers) and their implications for the successful establishment of agrarian communities in north-central Europe and for the development of subsequent cultures in the Middle and Late Neolithic remain fundamental questions in connection to that stage of the Neolithic settlement of Kuyavia. Two of these issues may be addressed through archaeogenetic research. This paper presents data on mtDNA haplogroup variation in BKG skeletal series from main archaeological sites of this cultural unit in Kuyavia, dated to 4700/4600–4100/4000 cal. BC.

### The Sites

**Fig 2:**
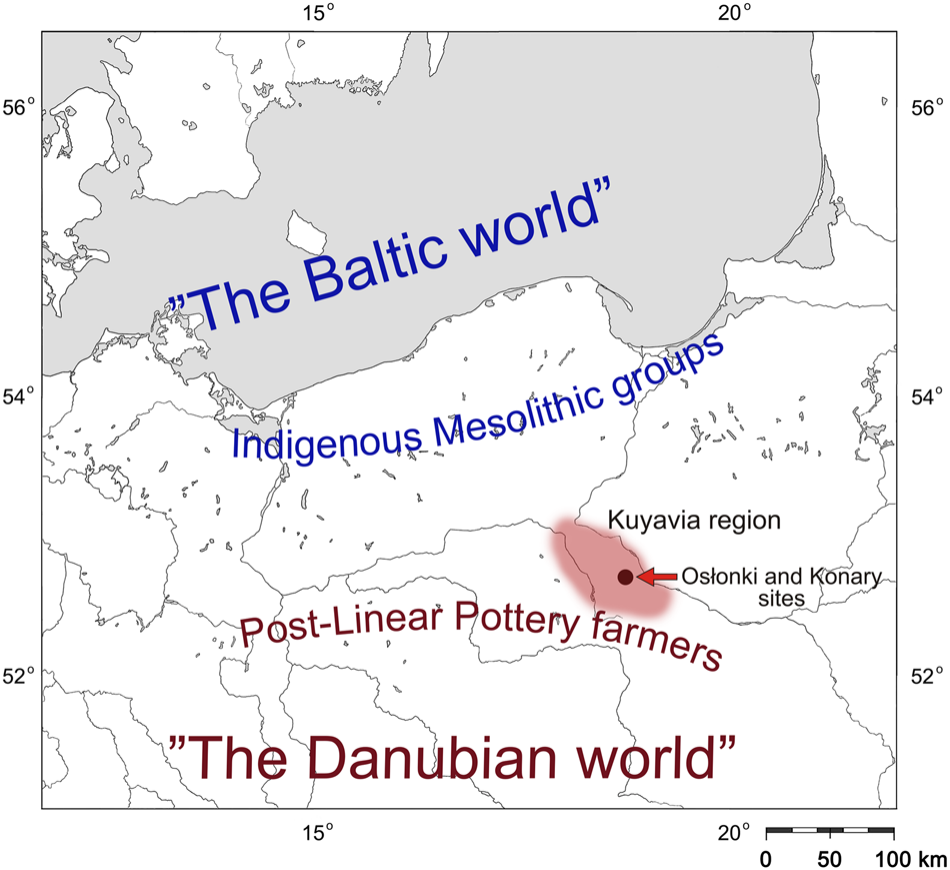
Map showing the location of the Kuyavia region and discussed sites in the north-central Poland. The names of cultural units outline the approximate edges of the territories populated by indigenous foragers populations and Neolithic farmers during the fifth millennium BC (after: [23]).

The most significant findings of the BKG were made at archaeological sites located in Brześć Kujawski (the eponymous site of this culture) and its immediate surroundings (within a radius of about 10 km from this town) (Fig. 2). The 183 skeletons representing the BKG that were discovered there constitute the largest culturally and spatially homogeneous collection of Neolithic skeletons in Poland, and also the oldest skeletal series of such magnitude in the country. All of the above-mentioned sites are relics of settlements (some of which were coexistent and formed settlement systems) with graves located within their confines. Exploration of the BKG in this area was initiated in the 1930s by the discovery of the Brześć Kujawski site [30]. The most extensive interdisciplinary studies of the sites were conducted by the Museum of Archaeology and Ethnography in Łódź from 1976 to 2004 (with some intervals) [27, 31]. The archaeological results for those sites were published in a number of papers [32–35], and the BKG has become a model example of adaptation of the Danubian Neolithic to the biogeographic environment of the North European Plain.

## Materials and Methods

Human skeletons from the BKG archaeological sites are part of the collection of the Museum of Archaeology and Ethnography in Łódź. The skeletons are currently on loan to the Department of Anthropology and stored in its osteological depository located in the building of the Faculty of Biology and Environmental Protection, University of Łódź (in the city of Łódź). The possession and analysis of the skeletal samples were in accordance with the legal status of archaeological human remains in Poland [36]. No permits were required for the described study, which complied with all relevant regulations.

The archaeogenetic study involved 25 best-preserved skeletons from four sites: site 1 in Osłonki (excavated in 1989–1994, graves no. 10, 11, 19, 23, 24, 25, 26, 38, 40, 50, 53, 57, 60, 61, 63, 67, 70, 75, 76, 80, 81), site 4 in Brześć Kujawski (excavated in 1933–1939, grave no. 29) and sites 1 and 1a in Konary (excavated in 1998–1999, graves no. 3, 5, 10). The sites are relics of a settlement system consisting of a large central settlement, (Osłonki site) and satellites (both sites in Konary). Skeletons from these sites are very well preserved thanks to the slightly alkaline pH of the soil and the presence of calcium carbonate [31]. Such conditions are conducive to isolation of authentic DNA sequences [37]. A total of 30 samples were taken from 25 individuals (five individuals were sampled twice); 28 samples consisted of teeth (26 permanent and 2 deciduous teeth) and 2 samples were fragments of the cortical bone of the femur shaft. The selected teeth were all well-preserved, free of decay, heavy attrition affecting dentine, or marked cracks in the enamel. Furthermore, the teeth were extracted as whole in such a way as not to damage the roots, the presence of which facilitated removal of contemporary contaminations from their surface.

### DNA extraction

The tooth samples taken from the selected skeletons were delivered in sterile containers to the aDNA laboratory at the Department of Molecular Biology, Medical University of Łódź, and frozen until the beginning of the isolation procedure. Mechanical cleaning of each tooth with a Dremel tool was followed by washing in NaClO for 30 min. and intensive rinsing in 96% ethanol. Exposure of each side of a tooth to UV light for 30 min was followed by tooth grinding in a freezer mill (SPEX SamplePrep 6770), and typically 0.3 to 0.6 g of tooth powder was incubated with 0.5 M EDTA (pH = 8.0) for 48 h. After decalcification, the sample was incubated for further 2 h at 56°C with proteinase K and *N*-phenacylthiazolium bromide (PTB). The obtained solution was submitted to DNA isolation in a MagNA Pure Compact Nucleic Acid Purification System (Roche), according to the manufacturer’s instructions. The obtained DNA was quantified (Qubit 2.0, Invitrogen or Eco Real-time PCR System, Ilumina), and amplified within 24 hours.

### Mitochondrial DNA analysis

The hypervariable region I (HVR-I) (16112–16380) was amplified using two primer pairs (S1 Table): L16112 (5’-CGTACATTACTGCCAGCC-3’), H16262 (5’-TGGTATCCTAGTGGGTGAG-3’) and L16251 (5’-CACACATCAACTGCAACTCC-3’), H16380 (5’-TCAAGGGACCCCTATCTGAG-3’). Most of the sequences were readable between positions 16115 and 16340 of HVR-I and resulted from two overlapping PCR products (186 bp and 171 bp). HVR-I was amplified in 25 μL of reaction mixture with 3–4 μL of all standard reagents, including AmpliTaq Gold (Applied Biosystems), at annealing temperature of 54°C during 38 cycles. Purification on spin columns (PCR Clean-up, Macherey-Nagel) was followed by amplicon extension using the BigDye 3.1 termination-ready reaction mix (Applied Biosystems). Four μL of the BigDye mix, 30 ng of the primer and 50–70 ng of the amplicon were used for each sequencing reaction (20 μL). Initial denaturation at 95°C for 5 minutes was followed by 36 cycles (95°C for 30 seconds, 56°C for 8 seconds, and 60°C for 4 minutes). The extended products were purified on spin columns (ExTerminator, A&A Biotechnology), dried in a Speed-Vac system (Savant), resuspended in 20 μL of deionized formamide, and sequenced on an ABI Prism 3130 Genetic Analyzer (Applied Biosystems). Sequences were edited and analyzed using a BioEdit sequence editor and MEGA 4 software [38].

The status of the H and U haplogroups was typed by RFLP analysis using *Alu*I (np7025) and *Hinf*I (np12308). Restriction sites at both nucleotide positions (np) are indicative for the haplogroups, respectively, according to MITOMAP [39]. Mutation at np15607 was chosen as the one characteristic for haplogroup T based on PhyloTree build 16 [40], and status of this haplogroup was confirmed by sequencing of the fragment between np15499 and np15635. Haplogroups were identified based on HaploGrep algorithm [41].

### Additional DNA preservation analysis

Tooth powder obtained through grinding was incubated in 1 M HCl (300 mg in 5 mL of HCl) at 48°C for 5 h. The insoluble fraction of collagen (precipitated by centrifugation at 7000 × *g* for 5 min) was dried at 56°C for 18 h after several washings (until reaching neutral pH). Collagen content was calculated as the ratio of dry weight of the insoluble fraction to the initial weight of tooth powder. The fact that it exceeded 2% suggests a high likelihood of DNA molecule recovery [42].

### Authentication of DNA sequences

The authentication procedure was previously described by Witas at al. [37]. In short, the preparation step and molecular analysis were carried out by suitably trained personnel in a laboratory especially dedicated to work with ancient DNA. Multiple mock controls were carried out during DNA extractions from teeth of the examined individuals and performed by different laboratory workers. For five randomly selected individuals the entire procedure, from tooth extraction to DNA isolation, was carried out twice with about a month’s interval in order to verify the repeatability of results. As the studied material is extremely valuable and a limited number of well-preserved teeth was accessible, the procedure was not applied to all the studied individuals. Besides, it was not necessary because no inconsistencies were detected in aDNA data for the repeated samples. The authenticity of the analyzed sequences was verified by comparing them with the mtDNA sequences of all the workers involved in DNA processing. The multiparameter profile (mtDNA haplotypes and nuDNA sequences) of individual patterns provided precise information that would identify a contaminating staff member. The extraction of teeth from one specimen and the isolation of DNA from different, independently ground, powder portions were carried out by workers with different DNA profiles. Laborious and expensive cloning was replaced by sequencing multiple isolates from the same specimen, as suggested by Winters et al. [43], and successfully applied by Witas et al. [37] and others [44]. Usually two DNA isolates provided a consensus sequence (2 from each tooth). An additional tooth analysis was performed in case of a low initial number of copies. A loss of repeatability resulted in rejection of the sample from further analysis and the procedure was repeated using another tooth, if available.

### Statistical analysis

Analysis of mtDNA HVR-I sequence was performed using Arlequin 3.5 [45]. Genetic differences between the studied populations or the distance between populations (*F*_*ST*_) were estimated according to the formula of Reynolds [46], and P-values were based on 10,000 permutations. Additionally, a craniometric pattern of affinity between the analyzed BKG, Mesolithic, and Neolithic populations from central Europe was determined based on Euclidean distances. The obtained matrix of Euclidean distances was subjected to cluster analysis according to Ward’s method [47]. All calculations were made using Statistica 9 PL software [48].

## Results

An average value of collagen content in a sample amounted to 4.2 ± 1.9%, which is consistent with a rather low DNA yield of the analyzed Neolithic skeletal material. The efficiency of DNA isolation amounted to approx. 37%: 11 out of 30 samples yielded a reproducible HVR-I sequence. mtDNA sequence analysis led to identification of four mtDNA macrohaplogroups—H, U5, T, and HV0 (Table 1).

**Table 1:** Summary of the genotyping data in the analyzed mtDNA sample of the BKG.

Subject*	Age at death (years)	Sex	Coding sequence	HVR-I region 16115–16340	Haplogroup
K1, 10	35–45	f	7028C	CRS	H
K1a, 5	15–20	?	7028T 15607G	16126C, 16189C, 16294T, 16296T, 16304C	T2b
O, 10	35–45	m	7028C	CRS	H
O, 11	approx 8–10	?	7028C	16304C	H5
O, 26	35–45	m	7028T	16298C	HV0
O, 38	25–35	m	7028T 12308G	16256T, 16270T	U5a
O, 40	20–30	f	7028C	16304C	H5
O, 60	3040	m	7028T 15607G	16126C, 16294T, 16296T, 16304C	T2b
O, 63	approx 25–30	m	7028C	CRS	H
O, 70	14–16	?	7028C	16304C	H5
O, 75	approx 14–15	?	7028C	16189C	H1

CRS—Cambridge Reference Sequence.

*Subject: K1 and K1a—Konary site 1 and 1a, O—Osłonki site (subsequent digit stands for the grave number).

As the presented results are the first data for Neolithic populations from Poland, a comparative analysis of the identified haplotypes was performed using data published for Mesolithic and Neolithic populations from central Europe [17], that is, metapopulations of hunter-gatherers (HGC) and the LBK, as well as several populations from the Mittelelbe-Saale region in Saxony-Anhalt (Germany): the Early Neolithic Rössen culture (RSC) and Schöningen Group (SCG), the Middle Neolithic Baalberge culture (BAC), Salzmünde culture (SMC), and Bernburg culture (BEC), and the Late Neolithic Corded Ware culture (CWC) and Bell Beaker culture (BBC). In general, the haplogroups obtained for the BKG are typical of Neolithic populations from central Europe and form part of the “mitochondrial Neolithic package” [17]. Due to the small sample size, it would be difficult to provide a definitive interpretation of the haplogroup profile of the analyzed population, but of significance is the very high frequency of haplogroup H (almost 64%). Although this haplogroup is typical of Neolithic populations (as opposed to hunter-gatherers) and dominates their mtDNA profiles [18, 49, 50], it has not been found to be so widespread in any of them. The frequency of haplogroup H in the BKG is also higher than that in present-day Western Europeans, in whom it is twice as frequent as in Early Neolithic populations [18]. The identical hg H haplotypes (H5) of individuals from graves 11, 40, and 70 in Osłonki may also suggest that they were related in maternal lineage. This interpretation is additionally supported by the fact that all three graves date back to the middle period of the settlement (4500/4450 to 4300 BC). The stratigraphy of the relics of the longhouses with which these graves are associated would then suggest that the female from grave 40 is a descendant of the family to which the individual from grave 70 belonged. However, the three graves do not exhibit any common features suggesting that they belong to related individuals (but the absence of such characteristics does not preclude such an option, either). It is worth mentioning here that graves from the middle period of the Osłonki settlement are grouped into small cemeteries located near individual longhouses and consisting of several burials each. Therefore, they are probably family cemeteries belonging to the families inhabiting particular longhouses (although results from the Neolithic community of Çatalhöyük [51] show that such an intuitive interpretation of a burial pattern within a settlement is not necessarily correct). Against this background, it should be noted that the three burials in question are located in different groups of graves, in distant parts of the settlement. The burials also differ in terms of the richness of grave goods, which indicate an individual’s status, from very poor (grave 40), to average (grave 11), to very rich (grave 70). On the other hand, the Neolithic BKG communities were probably transegalitarian and social status was not inherited [52]. Given such a diverse set of information, it will not be possible to conclusively answer questions as to the relationships between the studied individuals and their potential effect on the high frequency of haplogroup H until further data concerning both mtDNA and nDNA are obtained.

In turn, haplogroup N1a, which is very widespread among the first farmers of central Europe (LBK), and is also present in almost all cultures of the Early and Middle Neolithic (RSC, SCG, BAC, and SMC; source: S9 Table in Brandt et al. [17]), was not found in the analyzed BKG sample. As it was already stated, due to the low number of analyzed individuals, this finding might be a consequence of a stochastic sampling error, but it should be emphasized that the number of BKG individuals without this haplogroup was even higher, as in five cases in which HVR-I fragments did not allow for haplogroup determination due to an insufficient number of amplifiable templates, N1a was ruled out as a result of the lack of mutations specific for this haplogroup (16147A 16172C 16223T 16248T 16320T). Another important haplogroup from the point of view of the interpretation of the obtained results is haplogroup U. It was found once in the analyzed BKG sample, which translates into a relatively high frequency (approx. 9%) given the small number of analyzed individuals. However, this fact is important in that haplogroup U occurs sporadically or is absent from the previously described populations representing the post-Linear Pottery cultural tradition (approx. 3.9% in the LBK, 0% in the RSC; Table S9 in Brandt et al. [17]), while it is a characteristic element of the genetic structure of Middle and Late Neolithic populations (due to a reflux of hunter-gatherer haplogroups from the north of the continent [17]) which formed separate cultural traditions after the end of the “Danubian World,” that is, the TRB with its local groups (BAC, SMC, BEC), the CWC, and the BBC. Nevertheless, it will not be possible to conclusively state whether the above differences in haplogroup frequencies between the BKG and the other Early and Middle Neolithic cultures in central Europe actually reflect a specific mitochondrial DNA profile of BKG populations, until the database for this archeological unit is expanded.

*F*_*ST*_ genetic distances between the analyzed BKG sample and selected Neolithic cultures and hunter-gatherers (S2 Table), obtained on the basis of HVR-I haplotypes, are presented in Table 2 (it should be noted that due to the analysis of a different HVR mtDNA range the *F*_*ST*_ genetic distances calculated in our study differ slightly from those presented by Brandt et al. (S6 Table in [17]). Statistically significant differences were found between the BKG and the hunter-gatherers (the same concerns almost all other Neolithic cultures included in the comparison) and the LBK. On the other hand, the BKG practically does not differ from the Early Neolithic RSC (the post-Linear Pottery cultural tradition) and the Middle Neolithic SMC (the Funnel Beaker culture horizon).

**Table 2:** *F*_*ST*_ distances between Mesolithic and Neolithic groups from central Europe based on HVR-I mtDNA sequence (abbreviations are listed in the legend of the Table).

Group	Mesolithic	Early Neolithic			Middle Neolithic				Late Neolithic	
	HGC	LBK	RSC	SCG	BKG	BAC	SMC	BEC	CWC	BBC
HGC	-	0.15376*	0.10917*	0.14275*	0.14436*	0.12052*	0.14874*	0.03556	0.06907*	0.04216*
LBK	0.15376*	-	0.00363	0.01034	0.06121*	0.00344	0.02504	0.03281	0.03714*	0.07453*
RSC	0.10917*	0.00363	-	0.00247	0	0	0	0	0.00459	0.02241
SCG	0.14275*	0.01034	0.00247	-	0.05366	0	0.00639	0.00082	0.1114	0.05676*
BKG	0.14436*	0.06121*	0	0.05366	-	0.12052*	0	0.01479	0.03028	0.02802
BAC	0.12052*	0.00344	0	0	0.12052*	-	0.01686	0	0	0.03004
SMC	0.14874*	0.02504	0	0.00639	0	0.01686	-	0.01447	0.01924	0.04562*
BEC	0.03556	0.03281	0	0.00082	0.01479	0	0.01447	-	0	0
CWC	0.06907*	0.03714*	0.00459	0.1114	0.03028	0	0.01924	0	-	0.01003
BBC	0.04216*	0.07453*	0.02241	0.05676*	0.02802	0.03004	0.04562*	0	0.01003	-

**HGC**, Hunther-Gatherers, central Europe; **LBK**, early Linear Pottery; **RSC**, Rössen culture; **SCG**, Schöningen group; **BKG**, Brześć Kujawski Group; **BAC**, Baalberge culture; **SMC**, Salzmünde culture; **BEC**, Bernburg culture; **CWC**, Corded Ware culture; **BBC**, Bell Beaker culture.

*P < 0.05

**Fig 3:**
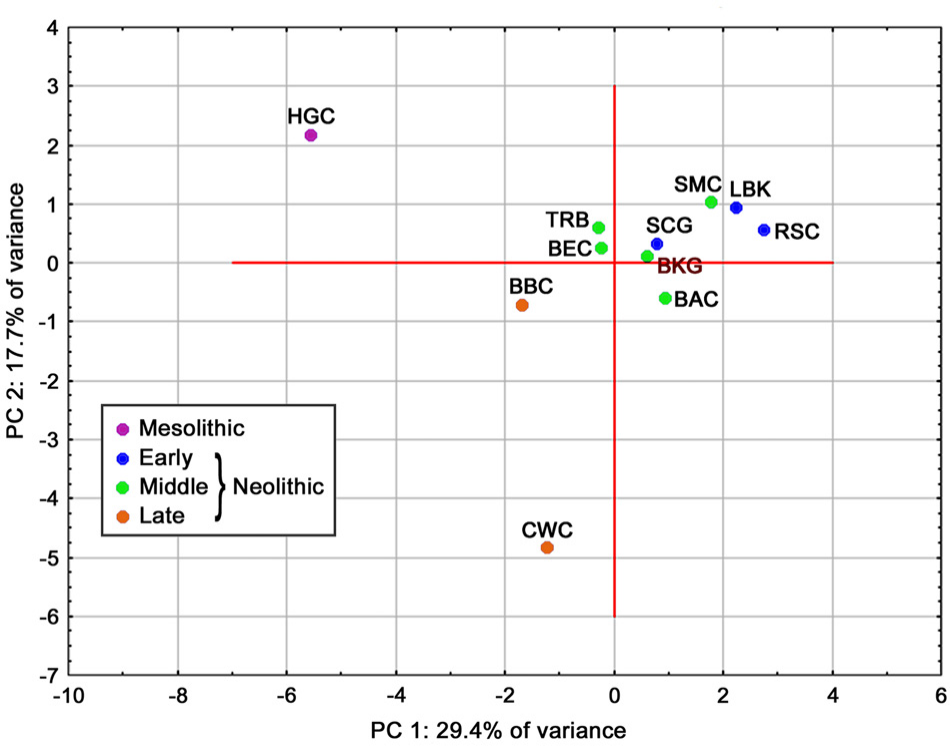
Plot of the first two principal co-ordinates illustrating patterns of affinity between the analyzed populations based on frequencies of mtDNA haplogroups (TRB—Funnel Beaker Culture; other abbreviations for Mesolithic and Neolithic cultural units as in the footnote of Table 2).

The BKG was also compared with the above-mentioned populations based on haplogroup frequencies, using principal component analysis (PCA). In this case, too, comparative data were taken from the Table S9 in the work of Brandt et al. [17], and additionally a skeletal series representing the TRB from Germany and Sweden was included. The first two principal components account for 47.1% of the total genetic variance in the compared populations, while the first three components account for 60.3% (S3 Table). The other components explain a small proportion of the variance, and so were omitted from the analysis. The scatter plot of the first two principal components (Fig. 3) forms one major cluster comprising all Early and Middle Neolithic cultures. This cluster could be further subdivided into two groups: one containing the LBK, RSC, and SMC, and the other composed of the BKG, SCG, TRB, as well as the BAC and BEC (associated with the southern group of the Funnel Beaker culture). The Late Neolithic BBC and CWC as well as hunter-gatherers remained outside of this cluster. Detailed analysis of the principal components shows that the first one primarily involves differences due to the frequency of N1a and most U haplogroups (which gave rise to the above-mentioned separation of Early and Middle Neolithic populations from those with hunter-gatherers lineages), the second one concerns haplogroups I, T1, and U2, while the third one is mostly associated with the Neolithic haplogroups HV, H, J, and K (S4 Table).

## Discussion

Application of genetic methods in studies on the Neolithization of Europe primarily aimed at explaining the beginnings of the process—whether it proceeded through colonization by farming populations or through the adoption of domesticated plants and animals by indigenous foragers. Currently, the focus shifts towards the period following the spreading of first farmers to reconstruct the population processes which have been occurring since Mesolithic foragers and early Neolithic farmers until present-day Europeans, carrying fundamentally different genetic profile.

In the archeological literature, the LBK and subsequent post-Linear Pottery units of central Europe are regarded as a single cultural tradition [53], closely linked to the Carpathian Basin, and contrasted with the later cultures of the Middle and Late Neolithic, such as the TRB or the Globular Amphora culture. The emergence of the TRB marks the beginning of the second stage of Neolithization of the northern part of central Europe, which involved populations of indigenous foragers to a much greater extent than before [28] as confirmed by the results of recent archaeogenetic studies [17]. On the other hand, in the light of these data, the Linear Pottery cultural tradition seems to diverge into a phase linked to the LBK, with mtDNA lineages showing affinity with present-day Near Eastern and Anatolian populations, and a post-LBK phase, with lineages more similar to present-day Central Europeans [18]. However, according to Brandt et al. [17] there was a genetic continuity between the LBK and the subsequent Early and Middle Neolithic cultures which lasted over 2500 years after the introduction of farming, that is, until 3000 BC.

The question arises as to the position of the studied BKG population from central Poland against the backdrop of the above-mentioned changes. Given the values of *Fst* genetic distances (Table 2), in general the BKG shows some similarity to populations representing the post-Linear Pottery cultures, and especially the RSC (except for the N1a and U5a haplogroups, which seem to have played a major role in that period), while it significantly differs from the LBK. On the other hand, the BKG also shares an affinity with the Middle Neolithic cultures associated with the Funnel Beaker culture: in the plot of principal components based on haplogroup frequencies the analyzed population is found in the cluster consisting mostly of the TRB and related cultural units (Fig. 3). As already stated, the basic problem with the interpretation of the obtained results is the small size of the sample. Thus, an important issue is whether the resulting interpopulation patterns reflect actual population relationships between the analyzed Early and Middle Neolithic cultures. Indeed, it seems to be so, as the presented analyses are corroborated by previous archeological [27] and anthropological studies [54] of the BKG. The Brześć Kujawski and Osłonki region in Kuyavia has an exceptional archaeological record, which makes it possible to reconstruct the history of its settlement since the emergence of the first LBK farmers and to elucidate the cultural and chronological relationships between the human groups inhabiting that area. Archeological findings clearly show a discontinuity in settlement at the beginning of the 5th millennium BC between the LBK and subsequent post-Linear Pottery cultures (in particular the Stroke-Ornamented Pottery culture). Secondly, many preserved artifacts suggest the contribution of groups from the Mittelelbe and Saale region (and especially the Rössen Culture) to the rise of the BKG [27]. As can be seen, the results of the presented paleogenetic study correspond well with the above data.

Of much greater interest is the potential relationship of the BKG with the Funnel Beaker culture complex, which played a key role in the Neolithization of northern Europe, as Kuyavia was once believed by some archaeologists to be the cradle of this culture, and some associations were suggested between the later Danubian cultures and the early TRB [55–57]. In terms of the obtained haplotypes, the BKG is similar to both the TRB-related archaeological units in the Mittelelbe-Saale region and the TRB skeletal series from Germany and Scandinavia (Fig. 3). Even though according to Price [58] the transition to agriculture in southern Scandinavia is likely to have occurred as a result of local hunters adopting the new mode of subsistence rather than through colonization by farmer groups from the south, the haplogroup results reported to date point to relatively large genetic differences between those two groups in Scandinavia [59] and to an affinity of TRB populations from that region to the central European LBK and the extant populations of Mediterranean Europe [60, 61]. On the other hand, the persistence of the Mesolithic substrate in southern Scandinavia is corroborated by, e.g., the pronounced hunter-gatherer-related admixture in Neolithic farmers [59] and the high frequency of haplogroup U in Late Neolithic and Bronze Age Denmark [62].

**Fig 4:**
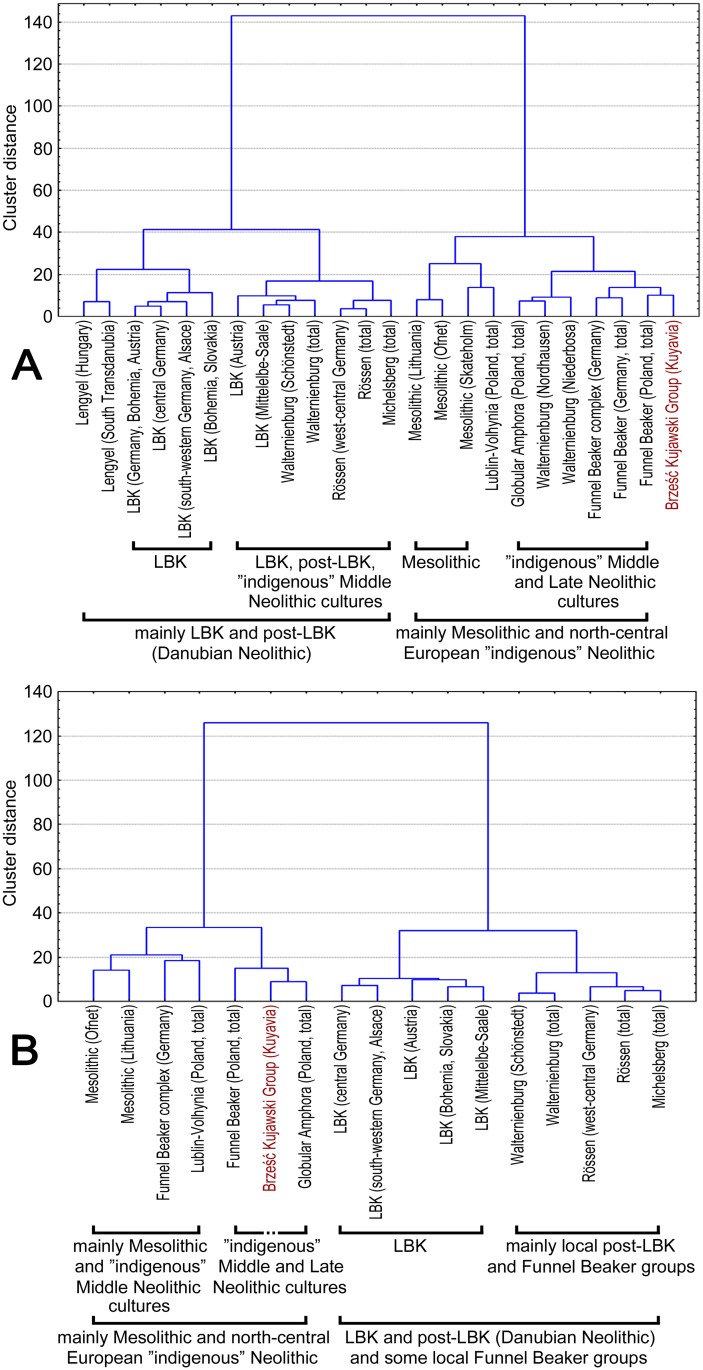
Affinities between the Mesolithic and Neolithic populations from central Europe based on craniometric data: male (A) and female (B) series.

Another limitation on the interpretation of the presented results is the absence of comparative data for populations inhabiting regions close to the BKG as this work is the first study of mtDNA lineages of Neolithic populations from the area of present-day Poland. This is not to imply that Neolithic skeletal series are scarce in this region in general. However, most of them were found in excavations carried out a long time ago, which significantly reduces the possibility of obtaining aDNA due to archeological preservation and storage conditions. On the other hand, the literature provides morphological characteristics for most of those series, at least in the form of arithmetic means of traditional metric traits. These data were used here to compare the analyzed BKG with 23 cranial series representing Mesolithic and Neolithic populations from central Europe (S5 Table). The craniometric data consisted of ten standard caliper measurements (S6 Table). The calculations were based on arithmetic means using Euclidean distance adopted as a measure of biological distance. The choice of arithmetic means over individual measurements was dictated by the fact that only such data were available for many of the compared skeletal series. A matrix of Euclidean distances was used to perform Ward clustering separately for male and female series (Fig. 4). As can be seen, the analyzed skeletal series are clearly grouped according to cultural and chronological categories, despite the use of very simple statistical methods. This indicates considerable morphological differences between human populations representing different Mesolithic and Neolithic cultural units. Moreover, the obtained results correspond to the main elements of mtDNA lineage variation in the studied populations (as described in this paper and quoted from the literature). First, LBK populations are clearly distinct and form a cluster irrespective of their local affinities, and second, Middle Neolithic and Late Neolithic populations had a tendency to merge with Mesolithic populations, which is reflected in the increased frequency of hunter-gatherer mtDNA haplotypes in that period, as reported by Brandt et al. [17]. On the other hand, the territorial affinities of the analyzed skeletal series are also visible, as exemplified by the separate cluster formed by populations from the Mittelelbe-Saale region, and especially by female groups. In the case of the BKG, the use of a broader comparative background weakened its affinities with the RSC, while emphasizing its similarity to cultures from the TRB culture complex. Interestingly, also Mesolithic populations are found in clusters with the BKG and TRB. Generally, cranial morphology puts the BKG among cultures which emerged following the end of the Linear Pottery tradition and indicates some contribution of post-Mesolithic populations. The pattern of relationships between the analyzed populations resulting from cranial traits is also important in that it concerns both males and females, in contrast to mtDNA haplogroups, which represent only maternal lineages.

Analyzing the relationship of the BKG to Mesolithic groups and the Neolithic cultures that are thought to have absorbed the post-Mesolithic substrate, one should also take into consideration the paleogenetic results which were recently presented by Gamba et al. [63] for early farmers in the Great Hungarian Plain, 6000–5000 cal BC. The first farmers in this region, representing early Neolithic cultures like Körös and LBK, incorporated local hunters-gatherers into their communities, which can be seen in the mtDNA and Y-chromosome haplogroups.

In summary, the analyzed BKG population markedly differs from the first farmers in Central Europe representing the LBK, which is part of a broader phenomenon probably caused by the crisis of agricultural communities in this area between the early and late stages of the Linear Pottery culture tradition [24–26]. This was previously described for populations in the Mittelelbe-Saale region by Brotherton et al. [18], who suggested that mtDNA lineages characteristic of the Early Neolithic LBK were most probably superseded between 4100 and 2200 BC. In the case of Kuyavia, indications of this population discontinuity were first provided by archaeological [27] and anthropological data revealing major changes in cranial morphology [54]. The paleogenetic findings presented in this paper additionally suggest that in north-central Poland this genetic discontinuity was more pronounced than in the Mittelelbe-Saale region: while being a post-Linear Pottery culture, the BKG exhibited a considerable F_*ST*_ distance from the LBK similarly as the Late Neolithic BBC and CWC, which represented totally different cultural traditions. Furthermore, it can be inferred that the discussed change in the genetic structure of Neolithic populations in north-central Poland occurred earlier (in terms of absolute chronology) than suggested by Brotherton et al. [18] or Brandt et al. [17]: nine out of the 11 individuals for which haplogroups were determined (including the individual with haplogroup U5a) come from BKG settlement stage in Kuyavia, which is dated to approx. 4600/4500 to 4300 BC. Of great importance is also the relationship between the BKG and the TRB in terms of mtDNA haplotypes (and even more distinctly in cranial morphology), referring to the hypothesis that central Poland was the cradle of this culture, which played a key role in the Neolithization of northern Europe. On the other hand, this finding might also be explained by the absorption of autochthonous Mesolithic groups by the Neolithic farmer communities present in Kuyavia following the decline of the LBK. However, to provide a more definitive answer to this question, it would be necessary to conduct further research into the mtDNA of human populations inhabiting north-central Poland at that period.

## Supporting Information

S1 Table Amplified mtDNA fragments, sequence of primers and PCR conditions. (DOCX)

S2 Table List of HVR-I haplotypes estimated for different cultures and used in the statistical analysis. (DOCX)

S3 Table Eigenvalues and variation explained by the successive principal components. (DOCX)

S4 Table Eigenvectors of the correlation matrix (Fst matrix). (DOCX)

S5 Table Sources of craniometric data used to perform the Ward clustering (Fig. 4). (DOCX)

S6 Table List of craniometric measurements employed and data for the BKG series. (DOCX)
