# Supplementary material for: Between the Baltic and Danubian Worlds: The Genetic Affinities of a Middle Neolithic Population from Central Poland
Source: PLoS One. 2015 Feb 25;10(2):e0118316. doi: 10.1371/journal.pone.0118316 (PMC4340919; doi:10.1371/journal.pone.0118316)
Supplement: S1 Table — (DOCX) [file pone.0118316.s001.docx]

**Table S1.** Amplified mtDNA fragments, sequence of primers and PCR conditions.

| **mtDNA fragment** | **Primer pairs** | **Product length** | **Annealing temp. ˚C** |
| --- | --- | --- | --- |
| HVR I  (16112-16262) | 5’-CGTACATTACTGCCAGCC-3’  5’-TGGTATCCTAGTGGGTGAG-3’ | 186 bp | 55 |
| HVR I  (16251-16380) | 5’-CACACATCAACTGCAACTCC-3’  5’-TCAAGGGACCCCTATCTGAG-3’ | 168 bp | 55 |
| 6924 - 7078 | 5’-GCCCTAGGATTCATCTTTCT-3’  5’-CCTCCTATGATGGCAAATAC-3’ | 155 bp | 54 |
| 12216 - 12338 | 5’-CACAAGAACTGCTAACTCATGC-3’  5’-ATTACTTTTATTTGGAGTTGCACCAAGATT-3’ | 153bp | 54 |
| 15499 - 15635 | 5’-CGACCCAGACAATTATACCC-3’  5’-ATAGTAATAGGGCAAGGACG-3’ | 137bp | 54 |
