# Supplementary material for: Between the Baltic and Danubian Worlds: The Genetic Affinities of a Middle Neolithic Population from Central Poland
Source: PLoS One. 2015 Feb 25;10(2):e0118316. doi: 10.1371/journal.pone.0118316 (PMC4340919; doi:10.1371/journal.pone.0118316)
Supplement: S2 Table — (DOCX) [file pone.0118316.s002.docx]

**Table S2.** List of HVR-I haplotypes estimated for different cultures and used in the statistical analysis.

| **Culture** | **Haplotype** | **Number of haplotypes** |
| --- | --- | --- |
| Baalberge | CRS | 5 |
| n = 19 | 16126C 16292T 16294T 16296T | 1 |
| [1, 2] | 16126C 16163G 16186T 16189C 16294T | 1 |
|  | 16224C 16311C | 2 |
|  | 16189C 16192T 16270T | 1 |
|  | 16189C 16223T 16278T | 1 |
|  | 16147A 16172C 16223T 16248T 16320T 16325C | 1 |
|  | 16126C 16292T 16294T | 1 |
|  | 16179T 16189C 16223T 16255A 16278T T16297C | 1 |
|  | 16126C 16311C | 1 |
|  | 16311C | 1 |
|  | 16126C 16192T 16294T 16304C | 1 |
|  | 16126C 16153A 16294T | 1 |
|  | 16126C 16129G | 1 |
| Bell | CRS | 7 |
| Beaker | 16192T 16256T 16270T 16291T | 4 |
| n = 29 | 16293G | 1 |
| [1, 2] | 16126C 16294T 16324C | 1 |
|  | 16223T C16292T | 2 |
|  | 16126C 16140C 16163G 16186T 16189C 16294T | 1 |
|  | 16192T 16270T | 1 |
|  | 16179T | 2 |
|  | 16304C | 3 |
|  | 16126C 16153A 16294T 16296T | 1 |
|  | 16189C 16213A 16270T | 1 |
|  | 16189C | 1 |
|  | 16126C | 1 |
|  | 16256T | 2 |
|  | 16224C 16311C | 1 |
| Bernburg | CRS | 3 |
| n = 17 | 16189T C16192T 16223T C16270T 16311C 16336A | 1 |
| [1] | 16224C 16311C | 3 |
|  | 16126C 16294T 16296T 16304C | 2 |
|  | 16192T 16256T 16270T | 2 |
|  | 16223T 16292T | 1 |
|  | 16192T | 1 |
|  | 16304C | 1 |
|  | 16239T 16270T | 1 |
|  | 16223T 16189T 16278C | 1 |
|  | 16297C 16298C | 1 |
| Corded | CRS | 9 |
| Ware | 16189C | 1 |
| n = 44 | 16192T 16256T 16270T | 2 |
| [1-3] | 16224C 16311C | 2 |
|  | 16192T 16292T 16325C | 1 |
|  | 16256T 16270T | 1 |
|  | 16126C | 4 |
|  | 16189T 16223T 16278T | 3 |
|  | 16126C 16294T 16324C | 1 |
|  | 16145A T16224C 16311C | 1 |
|  | 16221T | 1 |
|  | 16189C 16192T 16270T | 1 |
|  | 16224C 16311C 16319A | 3 |
|  | 16126C 16163G 16186T 16189C 16294T | 1 |
|  | 16304C | 1 |
|  | 16126C 16294T 16304C | 2 |
|  | 16134T | 1 |
|  | 16129C 16189C | 1 |
|  | 16126C 16294T 16296T | 1 |
|  | 16126C 16163G 16186T 16294T | 1 |
|  | 16192T 16256T 16270T 16291T | 1 |
|  | 16126C 16163G 16186T 16189C 16294T | 1 |
|  | 16192T 16270T | 1 |
|  | 16298C 16311C | 1 |
|  | 16192T | 1 |
|  | 16126C 16292T 16294T | 1 |
| Rössen | CRS | 3 |
| n= 11 | 16126C 16153A 16294T 16296T | 1 |
| [1, 2] | 16298C 16311T | 1 |
|  | 16179T 16189T 16223T 16255A 16278T 16297C | 1 |
|  | 16224C 16311C | 1 |
|  | 16304C | 1 |
|  | 16126C 16189T 16294T 16296T | 1 |
|  | 16298C | 1 |
|  | 16147A 16172C 16223T 16248T 16320T | 1 |
| Salzmünde | CRS | 7 |
| n= 29 | 16126C 16294T 16296T 16304C | 2 |
| [1, 2] | 16304C | 5 |
|  | 16298C | 1 |
|  | 16311C | 1 |
|  | 16224C 16311C 16319A | 1 |
|  | 16126C | 4 |
|  | 16126C 16274A | 1 |
|  | 16189C 16223T 16278T | 1 |
|  | 16129A 16147A 16154C 16172C 16209C 16223T 16248T 16320T | 1 |
|  | 16224C 16311C | 1 |
|  | 16147A 16154C 16172C 16223T 16248T 16320T | 1 |
|  | 16126C 16193T | 1 |
|  | 16224C 16245T 16311C | 1 |
|  | 16270T 16304C | 1 |
| Schöningen | CRS | 7 |
| n=33 | 16224C 16293C 16311C | 2 |
| [1, 2] | 16224C 16311C | 8 |
|  | 16126C | 4 |
|  | 16126C 16193T 16278T | 1 |
|  | 16223T 16292T | 3 |
|  | 16147A 16154C 16172C 16209C 16248T 16320T | 1 |
|  | 16223T 16189T | 1 |
|  | 16192T 16270T 16304C | 1 |
|  | 16189T 16270T 16304C | 1 |
|  | 16126C 16292T 16294T | 1 |
|  | 16126C 16189T 16294T 16296T | 1 |
|  | 16189T 16221T 16234T 16290T 16324C | 1 |
|  | 16126C 16294T 16296T 16304C | 1 |
| Hunther-gatherer | CRS | 17 |
| n = 60 | 16189C | 4 |
| [4-10] | 16192T 16270T | 9 |
|  | 16114A 16192T 16256T 16294T | 1 |
|  | 16270T | 5 |
|  | 16192T 16256T 16270T | 2 |
|  | 16189C 16270T | 1 |
|  | 16189C 16265G 16270T | 1 |
|  | 16174T 16189C 16192T 16270T 16311C | 1 |
|  | 16223T | 2 |
|  | 16222T 16261T | 1 |
|  | 16189C 16192T 16270T | 1 |
|  | 16192T 16256T 16270T 16294T | 1 |
|  | 16223T 16189C 16298C 16325C 16327T | 3 |
|  | 16129C 16189C | 2 |
|  | 16192T 16256T 16270T 16318G | 1 |
|  | 16235G 16311C | 1 |
|  | 16256T 16270T | 3 |
|  | 16260T | 1 |
|  | 16311C | 2 |
|  | 16298C | 1 |
| LBK | CRS | 15 |
| n=102 | 16147A 16172C 16223T 16248T | 1 |
| [1, 2, 11, 12] | 16147A 16172C 16223T 16248T 16320T | 3 |
|  | 16311C | 5 |
|  | 16224C 16311C | 16 |
|  | 16298C | 5 |
|  | 16126C 16294T 16296T 16304C | 5 |
|  | 16147A 16154C 16172C 16223T 16248T 16320T | 1 |
|  | 16223T C16292T | 2 |
|  | 16126C | 10 |
|  | 16126C 16147T 16294T 16296T T16297C 16304C | 2 |
|  | 16126C 16189C 16294T 16296T | 3 |
|  | 16256T 16270T | 1 |
|  | 16147A 16223T 16248T 16320T | 1 |
|  | 16126C 16294T 16296T 16304C | 4 |
|  | 16126C C16292T 16294T 16296T | 2 |
|  | 16147A 16172C 16223T 16248T | 1 |
|  | 16129G | 1 |
|  | 16129G 16147A 16154C 16172C 16223T 16248T 16320T | 3 |
|  | 16189C 16223T 16278C | 1 |
|  | 16129G 16147A 16154C 16172C 16223T 16248T 16320T | 1 |
|  | 16224C 16249C 16311C | 3 |
|  | 16147A 16172C 16223T 16248T | 1 |
|  | 16223T C16292T | 1 |
|  | 16126C 16274A | 1 |
|  | 16192T 16256T 16270T | 1 |
|  | 16209C 16224C 16311C | 1 |
|  | 16224C 16311C 16319A | 1 |
|  | 16126C 16153A 16294T 16296T | 1 |
|  | 16126C 16294T 16296T 16304C 16321T | 1 |
|  | 16192T 16270T | 1 |
|  | 16147A 16154C 16172C 16223T 16248T 16320T | 1 |
|  | 16126C 16292T 16294T | 1 |
|  | 16126C 16294T 16304C | 1 |
|  | 16286T 16304C | 1 |
|  | 16126C 16294T 16296T | 1 |
|  | 16319A | 1 |
|  | 16213A | 1 |

**References:**

1. Brandt G, Haak W, Adler CJ, Roth C, Szécsényi-Nagy A, et al. (2013) Ancient DNA reveals key stages in the formation of central European mitochondrial genetic diversity. Science 342: 257-261.

2. Brotherton P, Haak W, Templeton J, Brandt G, Soubrier J, et al. (2013) Neolithic mitochondrial haplogroup H genomes and the genetic origins of Europeans. Nat Commun 4: 1719.

3. Haak W, Brandt G, de Jong HN, Meyer C, Ganslmeier R, et al. (2008) Ancient DNA, Strontium isotopes, and osteological analyses shed light on social and kinship organization of the Later Stone Age. PNAS 105(47): 18226-18231.

4. Bramanti B, Thomas MG, Haak W, Unterlaender M, Jores P, et al. (2009) Genetic discontinuity between local hunter-gatherers and central Europe’s first farmers. Science 326: 137-140.

5. Fu Q, Mittnik A, Johnson PLF, Bos K, Lari M, et al. (2013) A revised timescale for human evolution based on ancient mitochondrial genomes. Curr Biol: 23(7): 553-559.

6. Chandler H, Sykes B, Zilhão J (2005) Using ancient DNA to examine genetic continuity at the Mesolithic-Neolithic transition in Portugal. In: Arias P, Ontañón C, García-Moncó Ce, editors. Actas del III Congreso del Neolitico en la Peninsula Iberica. Santander: Monografias del Istituto Internacional de Investigaciones Prehistóricas de Cantabria 1. pp. 781-786.

7. Hervella M, Izagirre N, Alonso S, Fregel R, Alonso A, et al. (2012) Ancient DNA from hunter-gatherer and farmer groups from Northern Spain supports a random dispersion model for the Neolithic expansion into Europe. PloS ONE 7(4): e34417.

8. Sánchez-Quinto F, Schroeder H, Ramirez O, Ávila-Arcos MC, Pybus M, et al. (2012) Genomic affinities of two 7,000-year-old Iberian hunter-gatherers. Curr Biol 22(16): 1494-1499.

9. Der Sarkissian C, Balanovsky O, Brandt G, Khartanovich V, Buzhilova A, et al. (2013) Ancient DNA reveals prehistoric gene-flow from Siberia in the complex human population history of North East Europe. PLoS Genet 9(2): e1003296.

10. Krause J, Briggs AW, Kircher M, Maricic T, Zwyns N, et al. (2010) A complete mtDNA genome of an early modern human from Kostenki, Russia. Curr Biol 20(3): 231-236.

11. Haak W, Balanovsky O, Sanchez JJ, Koshel S, Zaporozhchenko V, et al. (2010) Ancient DNA from European Early Neolithic farmers reveals their Near Eastern affinities. PLoS Biol 8(11): e1000536.

12. Haak W, Forster P, Bramanti B, Matsumura S, Brandt G, et al. (2005) Ancient DNA from the first European farmers in 7500-year-old Neolithic sites. Science 310: 1016-1018.
