# Supplementary material for: Between the Baltic and Danubian Worlds: The Genetic Affinities of a Middle Neolithic Population from Central Poland
Source: PLoS One. 2015 Feb 25;10(2):e0118316. doi: 10.1371/journal.pone.0118316 (PMC4340919; doi:10.1371/journal.pone.0118316)
Supplement: S3 Table — (DOCX) [file pone.0118316.s003.docx]

**Table S3.** Eigenvalues and variation explained by the successive principal components.

| **Value** | **Eigenvalue** | **% total variance** | **Cumul. eigenvalue** | **Cumul. %** |
| --- | --- | --- | --- | --- |
| 1 | 5,295167 | 29,41760 | 5,29517 | 29,4176 |
| 2 | 3,177610 | 17,65339 | 8,47278 | 47,0710 |
| 3 | 2,386921 | 13,26067 | 10,85970 | 60,3317 |
| 4 | 2,075715 | 11,53175 | 12,93541 | 71,8634 |
| 5 | 1,609133 | 8,93963 | 14,54455 | 80,8030 |
| 6 | 1,331551 | 7,39751 | 15,87610 | 88,2005 |
| 7 | 1,055840 | 5,86578 | 16,93194 | 94,0663 |
| 8 | 0,615864 | 3,42147 | 17,54780 | 97,4878 |
| 9 | 0,385352 | 2,14084 | 17,93315 | 99,6286 |
| 10 | 0,066848 | 0,37138 | 18,00000 | 100,0000 |
