# Supplementary material for: Between the Baltic and Danubian Worlds: The Genetic Affinities of a Middle Neolithic Population from Central Poland
Source: PLoS One. 2015 Feb 25;10(2):e0118316. doi: 10.1371/journal.pone.0118316 (PMC4340919; doi:10.1371/journal.pone.0118316)
Supplement: S4 Table — (DOCX) [file pone.0118316.s004.docx]

**Table S4.** Eigenvectors of the correlation matrix (*Fst* matrix).

| **Haplogroup** | **Eigenvectors** | | | | | | | | | |
| --- | --- | --- | --- | --- | --- | --- | --- | --- | --- | --- |
|  | **Fact.1** | **Fact.2** | **Fact.3** | **Fact.4** | **Fact.5** | **Fact.6** | **Fact.7** | **Fact.8** | **Fact.9** | **Fact.10** |
| **N1a** | 0,303224 | 0,155742 | 0,070372 | -0,295178 | 0,051197 | 0,131793 | 0,084530 | 0,343519 | 0,581252 | 0,290145 |
| **I** | -0,077071 | -0,503352 | 0,071225 | -0,119745 | 0,140146 | 0,062422 | 0,206016 | 0,200017 | -0,196315 | 0,085561 |
| **W** | -0,013782 | -0,079008 | 0,290849 | 0,329853 | -0,438953 | 0,347882 | -0,254029 | 0,114571 | 0,081730 | -0,145317 |
| **X** | 0,171093 | -0,214792 | -0,037819 | -0,445223 | -0,225175 | 0,075441 | -0,010533 | -0,547659 | -0,123938 | -0,232216 |
| **HV** | 0,284526 | 0,015122 | -0,360478 | -0,201841 | -0,008072 | -0,062196 | -0,116274 | 0,428652 | -0,333228 | -0,076448 |
| **V** | 0,237469 | 0,147196 | -0,093248 | -0,147870 | -0,126325 | 0,427098 | 0,513997 | -0,155233 | 0,074654 | -0,168731 |
| **H** | 0,156180 | -0,112088 | -0,475914 | 0,268468 | 0,059714 | 0,140238 | -0,319883 | -0,001253 | -0,041551 | -0,028743 |
| **T1** | -0,069803 | -0,476724 | -0,028156 | -0,175116 | -0,010782 | -0,098296 | -0,239238 | -0,189067 | 0,483284 | 0,121735 |
| **T2** | 0,251886 | -0,021367 | -0,013171 | 0,087940 | -0,101113 | -0,632730 | 0,289905 | -0,022036 | 0,139873 | 0,013089 |
| **J** | 0,132077 | 0,002906 | 0,470873 | 0,113315 | 0,412020 | -0,213572 | -0,050305 | 0,012842 | 0,027746 | -0,469642 |
| **U** | -0,348862 | 0,228587 | -0,024193 | -0,264501 | 0,045681 | 0,047986 | 0,060243 | 0,216884 | -0,096757 | 0,031791 |
| **U2** | -0,077071 | -0,503352 | 0,071225 | -0,119745 | 0,140146 | 0,062422 | 0,206016 | 0,200017 | -0,196315 | 0,085561 |
| **U3** | 0,125392 | 0,118586 | 0,135931 | -0,043633 | 0,613264 | 0,306238 | -0,187414 | -0,278202 | -0,084197 | 0,310884 |
| **U4** | -0,390578 | -0,074865 | -0,047384 | -0,121362 | 0,079147 | 0,182200 | 0,003404 | 0,240903 | 0,299381 | -0,455998 |
| **U5a** | -0,328651 | -0,074951 | -0,159279 | 0,348045 | -0,034667 | 0,008237 | 0,258884 | -0,145045 | 0,095438 | 0,366078 |
| **U5b** | -0,370470 | 0,221348 | 0,091540 | -0,111029 | -0,019215 | -0,066052 | 0,206268 | -0,150451 | -0,131579 | 0,023553 |
| **U8** | -0,219298 | 0,153442 | 0,084792 | -0,413687 | -0,188013 | -0,206703 | -0,413663 | -0,015032 | -0,066375 | 0,166554 |
| **K** | 0,189422 | -0,066136 | 0,498808 | 0,007226 | -0,306041 | 0,100577 | -0,005490 | 0,135339 | -0,232121 | 0,295868 |
