# Supplementary material for: Between the Baltic and Danubian Worlds: The Genetic Affinities of a Middle Neolithic Population from Central Poland
Source: PLoS One. 2015 Feb 25;10(2):e0118316. doi: 10.1371/journal.pone.0118316 (PMC4340919; doi:10.1371/journal.pone.0118316)
Supplement: S5 Table — (DOCX) [file pone.0118316.s005.docx]

**Table S5.** Sources of craniometric data used to perform the Ward clustering (Fig. 4A and B)

| **Skeletal series** | **Source of data** |
| --- | --- |
| 1) Lengyel culture (Hungary)  2) Lengyel culture (South Transdanubia)  3) Funnel Beaker culture (Germany, total) | after: Piontek J, Marciniak A (1990) Struktura antropologiczna a kulturowe strategie adaptacyjne populacji neolitycznych w Europie Środkowej. Seria Antropologia 16. Poznań: Wydawnictwo UAM. 100 p. |
| 4) LBK (Germany, Bohemia, Austria)  5) LBK (central Germany)  6) LBK (south-western Germany, Alsace)  7) LBK (Bohemia, Slovakia)  8) LBK (Austria)  9) Walternienburg culture (Schönstedt)  10) Michelsberg culture (total)  11) Rössen culture (total)  12) Rössen culture (west-central Germany)  13) Walternienburg culture (Niederbosa)  14) Walternienburg culture (Nordhausen) | Knussmann R, Knussmann R (1978) Die Skelettreste der Rössener und Michelsberger Kulturepoche. In: Schwidetzky I, editor. Die Anfänge des Neolithikums vom Orient bis Nordeuropa. Teil VIIIb, Anthropologie, Teil 2*.* Köln-Wien: Böhlau Verlag. 164-217. |
| 15) LBK (Mittelelbe-Saale) | Bach A (1978) Neolitische populationen im Mittelelbe-Saale-Gebiet. Weimarer Monographien zur Ur- und Frühgeschichter. Weimar: Museum für Ur- und Frühgeschichte Thüringens. 144 p. |
| 16) Walternienburg culture (total)  17) Funnel Beaker culture complex (Germany) | Schwidetzky I (1978) Neolithische und frühbronzezeitliche Menschenfunde aus der DDR. In: Schwidetzky I, editor. Die Anfänge des Neolithikums vom Orient bis Nordeuropa. Teil VIIIb, Anthropologie, Teil 2*.* Köln-Wien: Böhlau Verlag. 93-119. |
| 18) Mesolithic (Lithuania) | Česnys G, Butrimas A (2009) Reinventing Mesolithic skulls in Lithuania: Donkalnis and Spiginas sites. Acta Medica Lituanica 16(1-2): 1-8. |
| 19) Mesolithic (Ofnet) | Scheidt W (1923) Die eiszeitlichen Schädelfunde aus der Grossen Ofnet-Höhle und vom Kaufertsberg bei Nördlingen. München: J. F. Lehmanns Verlag. 112 p. |
| 20) Mesolithic (Skateholm) | Persson O, Persson E (1988) Anthropological report concerning the interred Mesolithic populations from Skateholm, Southern Sweden. Excavation seasons 1983-1984. In: Larsson L, editor. The Skateholm Project I. Man and environment. Acta Regiae Societatis Humaniorum Litterarum Lundensis LXXIX. Stockholm: Almqvist & Wiksell International. 89-105. |
| 21) Lublin-Volhynia culture (Poland, total) | 1) Kozak-Zychman W, Gauda E (1996) Materiały kostne z pochówków kultury wołyńsko-lubelskiej ceramiki malowanej na stanowisku 10 w Strzyżowie, gm. Horodło, woj. Zamość. Archeologia Polski Środkowowschodniej 1: 189-190.  2) Kozak-Zychman W, Gauda E (1997) Materiały antropologiczne z grobów kultury wołyńsko-lubelskiej ceramiki malowanej odkrytych na terenie woj. zamojskiego. Archeologia Polski Środkowowschodniej 2: 245-253.  3) Kozak-Zychman W, Maślanka Z (2005) Szczątki kostne z pochówków ludności kultury wołyńsko-lubelskiej ceramiki malowanej na stan. 26 w Strzyżowie, pow. Hrubieszów. Archeologia Polski Środkowowschodniej 7: 176-182.  4) Kozak-Zychman W. Szeliga M (2005) Wyniki dalszych badań antropologicznych szczątków kostnych z pochówków kultury wołyńsko-lubelskiej ceramiki malowanej na stan. 1C w Gródku, pow. Hrubieszów. Archeologia Polski Środkowowschodniej 7: 173-175.  5) Zakościelna A (2010) Studium obrządku pogrzebowego kultury lubelsko-wołyńskiej. Lublin: Wydawnictwo UMCS. 551 p. |
| 22) Globular Amphora culture (Poland, total) | 1) Henneberg M, Kaczmarek M, Szymandera W (1982) Charakterystyka grupy ludności kultury amfor kulistych, na podstawie analizy szczątków kostnych z Chodzieży. Przegląd Antropologiczny 48 (1-2): 131-143.  2) Dzierżykraj-Rogalski T (1958) Crania et alia ossa polonica. Cmentarzysko neolityczne w Stoku i Lesie Stockim. Materiały i Prace Antropologiczne 30: 1-70.  3) Kozak-Zychman W, Szeliga M (2003) Ludność kultury amfor kulistych na Lubelszczyźnie. Gdańsk: Międzynarodowa Konferencja Naukowa PTA. pp. 582-586.  4) Kozak-Zychman W, Trzaska A (2006) Materiały antropologiczne ze zniszczonych pochówków ludności kultury amfor kulistych na stanowisku 6 w Miętowie, pow. lubelski. Archeologia Polski Środkowowschodniej 8: 31-34. |
| 23) Funnel Beaker culture (Poland, total) | 1) Dzierżykraj-Rogalski T (1958) Crania et alia ossa polonica. Cmentarzysko neolityczne w Stoku i Lesie Stockim. Materiały i Prace Antropologiczne 30: 1-70.  2) Kapica Z (1975) Neolityczne szczątki kostne z grobowca kujawskiego nr 9 w Sarnowie, pow. Włocławek. Sprawozdania Archeologiczne 27: 55-64.  3) Kozak-Zychman W (2001) Materiały kostne z pochówków ludności kultury pucharów lejkowatych na stanowisku 3 w Pawłowie, pow. Sandomierz, woj. świętokrzyskie. Archeologia Polski Środkowowschodniej 6: 20-21.  4) Kozak-Zychman W (2007) Pochówki na cmentarzysku ludności kultury pucharów lejkowatych w miejscowości Święcica, pow. sandomierski, stan. 1. Archeologia Polski Środkowowschodniej 9: 49-52.  5) Kozak-Zychman W, Szeliga M (2005) Pochówki z cmentarzyska ludności kultury pucharów lejkowatych w Pawłowie, stan. 3, pow. Sandomierz (badania 2001-2003 r.). Archeologia Polski Środkowowschodniej 7: 33-40.  6) Wiercińska A (1967) Charakterystyka antropologiczna szkieletu ludzkiego z jamy 16 na stanowisku Pieczyska w Zawichoście, pow. Sandomierz. Rocznik Muzeum Świętokrzyskiego 4: 35-43. |
| 24) Brześć Kujawski Group | Own data: Table S6. |
