# Supplementary material for: Between the Baltic and Danubian Worlds: The Genetic Affinities of a Middle Neolithic Population from Central Poland
Source: PLoS One. 2015 Feb 25;10(2):e0118316. doi: 10.1371/journal.pone.0118316 (PMC4340919; doi:10.1371/journal.pone.0118316)
Supplement: S6 Table — (DOCX) [file pone.0118316.s006.docx]

**Table S6.** List of craniometric measurements employed and data for the BKG series

| **Measurement** | **Code**  **(Martin and Saller 1957)** | **BKG (arithmetic mean and number of individuals)** | |
| --- | --- | --- | --- |
|  |  | **Males** | **Females** |
| Maximum cranial length | g-op (M1) | 189.1 (37) | 181.1 (30) |
| Maximum cranial breadth | eu-eu (M8) | 138.3 (29) | 133.0 (26) |
| Least frontal breadth | ft-ft (M9) | 99.1 (49) | 95.2 (33) |
| Basi-bregmatic height | ba-b (M17) | 141.0 (15) | 135.5 (16) |
| Bizygomatic breadth | zy-zy (M45) | 133.2 (17) | 122.4 (14) |
| Nasoalveolar height | n-pr (M48) | 67.7 (19) | 62.6 (22) |
| Orbital breadth | mf-ek (M51) | 42.3 (19) | 40.2 (24) |
| Orbital height | spa-sbk (M52) | 33.0 (20) | 31.0 (24) |
| Nasal breadth | apt-apt (M54) | 24.9 (30) | 24.1 (27) |
| Nasal height | n-ns (M55) | 49.9 (19) | 45.5 (23) |

**References**

Martin R, Saller K (1957) Lehrbuch der Anthropologie in Systematischer Darstellung, mit Besonderer Berücksichtigung der Anthropologischen Methoden. Bd. I. Stuttgart: Fischer. 518 p.
